# An Island of Stability: Art Images and Natural Scenes – but Not Natural Faces – Show Consistent Esthetic Response in Alzheimer’s-Related Dementia

**DOI:** 10.3389/fpsyg.2013.00107

**Published:** 2013-03-07

**Authors:** Daniel J. Graham, Simone Stockinger, Helmut Leder

**Affiliations:** ^1^Department of Psychology, Hobart and William Smith CollegesGeneva, NY, USA; ^2^Department of Psychological Basic Research and Research Methods, Faculty of Psychology, University of ViennaVienna, Austria

**Keywords:** Alzheimer’s disease, dementia, face perception, esthetics, natural scenes, esthetic stability, art perception, memory

## Abstract

Alzheimer’s disease (AD) causes severe impairments in cognitive function but there is evidence that aspects of esthetic perception are somewhat spared, at least in early stages of the disease. People with early Alzheimer’s-related dementia have been found to show similar degrees of stability over time in esthetic judgment of paintings compared to controls, despite poor explicit memory for the images. Here we expand on this line of inquiry to investigate the types of perceptual judgments involved, and to test whether people in later stages of the disease also show evidence of preserved esthetic judgment. Our results confirm that, compared to healthy controls, there is similar esthetic stability in early stage AD in the absence of explicit memory, and we report here that people with later stages of the disease also show similar stability compared to controls. However, while we find that stability for portrait paintings, landscape paintings, and landscape photographs is not different compared to control group performance, stability for face photographs – which were matched for identity with the portrait paintings – was significantly impaired in the AD group. We suggest that partially spared face-processing systems interfere with esthetic processing of natural faces in ways that are not found for artistic images and landscape photographs. Thus, our work provides a novel form of evidence regarding face-processing in healthy and diseased aging. Our work also gives insights into general theories of esthetics, since people with AD are not encumbered by many of the semantic and emotional factors that otherwise color esthetic judgment. We conclude that, for people with AD, basic esthetic judgment of artistic images represents an “island of stability” in a condition that in most other respects causes profound cognitive disruption. As such, esthetic response could be a promising route to future therapies.

## Introduction

Alzheimer’s disease (AD) has devastating effects on many aspects of cognition including memory (e.g., Parasuraman and Haxby, 1993) and perception (e.g., Cronin-Golomb, 1995) systems. With 115 million people worldwide expected to develop dementia as a result of AD by 2050 due to demographic trends (Alzheimer’s Disease International, 2009), there is an urgent need to understand the nature of these deficits and to explore possible routes to therapy, perhaps via partially spared cognitive systems.

Recent evidence suggests that one promising avenue for approaching Alzheimer’s-related dementia is via esthetic perception. Halpern et al. (2008) reported that patients with early-stage AD show essentially the same degree of stability in esthetic judgment of paintings over a 2-week span compared to age-matched controls. Crucially, patients showed this performance despite performing at chance on an explicit memory test of the images, whereas controls performed well above chance on the explicit memory test.

This discovery coincides with a surge of research in esthetic perception from a variety of viewpoints and with diverse methodologies (for reviews, see: Leder et al., 2004; Graham and Redies, 2010; Bacci and Melcher, 2011; Chatterjee, 2011; Van de Cruys and Wagemans, 2011). Indeed, empirical and neuro-esthetic research is emerging as an important facet of visual perception and cognition. Coinciding with this basic research on esthetics, there are increasing attempts to show that interactions with art can lessen the severity of AD symptoms (e.g., Wald, 2003; Eekelaar et al., 2012). Programs such as the New York Museum of Modern Art’s MeetMe program use art viewing as a route to reducing disease severity, which has shown some promising indications (Rosenberg et al., 2010). In addition, there are increasing efforts to integrate artistic and esthetic experiences into long-term care. For example, Hearthstone Alzheimer’s Care in the United States has a number of long-term care facilities that are explicitly structured around interactions with art and music (http://thehearth.org).

However, beyond the pioneering work of Halpern et al. (2008) much remains unknown about esthetic perception in AD. Halpern et al. (2008) demonstrated that the representationality of artwork does not seem to affect esthetic stability. In particular, paintings deemed by the investigators to be “representational,” “quasi-representational,” and “abstract” did not show significantly different interactions with stability (though both AD patients and controls showed the same small differences in stability for abstract versus quasi-representational images). Therefore, artistic style appears not to be a primary factor in generating stability.

We could look at this latter finding from a different perspective and ask whether it is an indication that artistic creation in the painted medium is itself a key element for esthetic perception. In other words, perhaps one component of the seemingly spared capacity to judge esthetic value consistently is the ability to recognize an art image as such. Given that the images used in Halpern et al. (2008) were presented outside of the traditional context of fine art (e.g., a museum), viewers were left only with visual cues to the images’ handmade artistic visual quality (for research on context dependency in esthetics, see Kirk et al., 2009; Leder, 2013). Certainly, the lush fantasy of Alma-Tadema and the dramatic use of color in landscapes by Hopper (classified by Halpern et al., 2008, as representational), the surreal imagery of Kitaj (classified as quasi-representational), and the complete abstraction of Mondrian (classified as abstract), are powerful cues to the artificiality of the images. Therefore, one of our primary goals in the present study is to measure esthetic stability in AD for handmade artistic representations in comparison to photographs of the same content. We predicted that painted artwork would display greater stability compared to other classes of images for the AD group.

A second major goal of the present study is to test specific classes of image content, in both artistic and photographic representations. In particular, we are interested in the role of faces. From decades of research, it is now clear that the human brain devotes considerable resources to face perception (e.g., Haxby et al., 2000), and in many ways the human face is treated as a “special” type of stimulus vis à vis perception (e.g., Bruce and Young, 1998).

Importantly, faces appear to have particular relevance to the progression of AD. There is evidence that recognition of familiar scenes could be impaired more than the perception of familiar faces at the earliest stages of the disease (Cheng and Pai, 2010), implying that face deficits emerge more slowly over the progression of the disease. Also, Kurylo et al. (1996) found that AD patients performed well on a face-matching tasks (Benton Face Recognition Test) and were near the level of performance shown by controls.

However, there is active debate about how visual perception in general and face perception in particular are affected by AD. Indeed, there is great variability in the kinds of tests of visual perception that have been employed with AD patients, as well as conflicting findings on comparable tests (see Kirby et al., 2010). With regard to faces, the majority of research in AD patients concerns face memory (recognition) rather than face perception (e.g., gender judgment, after-effects, etc.).

If performance on an esthetic task for a group of faces primarily involves perceptual rather than memory systems, we are left with relatively little consistent evidence upon which to base a hypothesis for how specific content could affect the outcome of our experiments. On the other hand, our results using the innovative approach of Halpern et al. (2008) will potentially be of unique importance for understanding perception deficits in AD, especially given the conflicting findings regarding face perception in AD described above.

In addition to our goal of understanding how image content and art’s handmade quality affect esthetic perception in AD, we also set out to test whether individuals with more severe stages of AD still show esthetic stability. Therefore, we test whether stable esthetic evaluations can be preserved even under conditions of severe memory impairment. We also ask if there are performance differences for varied image content that depend on the stage of AD. We predicted that esthetic stability would indeed be observed in later-stage AD patients, and that it would manifest itself in much the same way as it does in controls.

## Materials and Methods

### Overview

In the present studies we employed methods similar to Halpern et al. (2008). Participants were asked to sort stimuli in rank-order according to how much they liked the stimuli esthetically, and they were also tested on their explicit memory for the images. The study compared a clinical population with a group of control participants. However, unlike Halpern et al. (2008) who used a small set of stimuli with no special consideration of content (aside from using unfamiliar material and not repeating main content) in our study we systematically compared different classes of paintings and content-matched photographs. We were especially interested to carefully test the possible effects of the presence of faces. Although the stimulus classes in Halpern et al. (2008) varied to some extent in terms of face content – e.g., representational images mostly contained faces, while abstract images did not – the delineation was not strict. For example the representational class also included an Audubon painting of a bird, and the quasi-representational class contained a mix of images with and without faces.

### Participants

Participants were recruited from two institutions in Vienna, Austria: SeneCura Sozialzentrum Purkersdorf (3002 Purkersdorf, Bahnhofstraße 2) and Caritas (1190 Wien, Hameaustraße 45–47). All participants (via authorized caregivers) as well as both institutions gave consent to conduct the studies. All participants had been diagnosed according to ICD-10 in both intuitions by authorized persons, and were administered the Mini-Mental State Exam (MMSE; Folstein et al., 1975) prior to our experiments. Participants did not have a prior diagnosis of depression. Participants were not art experts, and they did not have perceptual problems that would have impaired the perception of the artworks (see Procedures).

From an initial set of 22, two participants were excluded because they could not finish the testing, and two others were excluded because they had missing values in the preference rank tasks. Data of 18 AD participants were therefore included in the analyses. Fifteen were female, and age varied between 74 and 97 years (*M* = 89.5, SD = 5.9). Art interest varied between 0 (“no interest at all”) and 11 (“great interest”), with a mean of 5.83 (SD = 2.18). The mean score on the MMSE was 15.56 (SD = 5.79) with a range from 7 to 25. We split the variable AD into three levels, following the procedure by Wijk et al. (1999), with severe AD indicated when MMSE was below 12 (7 participants), moderate AD with a range of 13–19 (5 participants), and early stage, with values between 20 and 26 (6 participants).

The control consisted of a group healthy older adults who all achieved values of 27 and above on the MMSE (Max. possible value = 30). Control participants were recruited from the same two institutions and from the wider community. Controls were roughly matched with the experimental group according to age and self-indicated interest in art. Also, it was assured that no artists were included in the sample.

The 15 participants in the control group (10 female, 5 male) had an average age of 74.2 (SD = 13.2) with a range between 59 and 99 years. All control participants had MMSE values at least 27 (*M* = 28.1, SD = 0.96). The art interest varied between 0 and 10 (“very much interested in art”), with a mean very similar to that of the AD patients (*M* = 5.4, SD = 3.1).

### Stimuli

**Figure 1:**
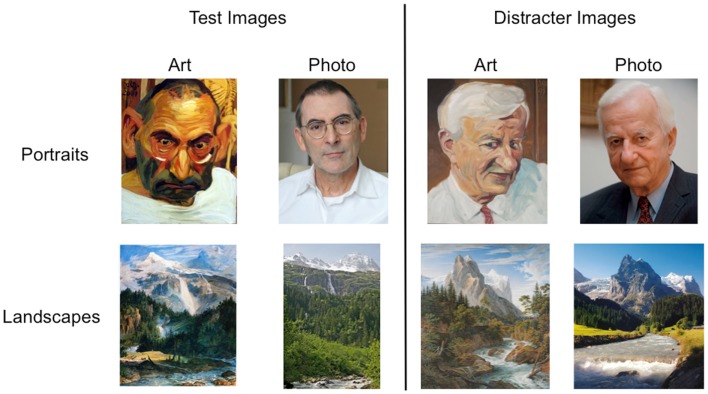
**Examples of stimuli used in the esthetic stability test (test images: P1, L5) and matched distracter images used in the explicit memory test (distracter images)**. Image metadata are provided in Table 1.

Four sets of eight images were used as stimuli (see Figure 1). We used images of the following types: “painted portraits”; “photographic portraits”; “painted landscapes”; and “photographic landscapes.” Images were printed in color on 185 cm × 135 cm high-quality photo paper. All artworks were of recognizable content. They were painted in representational style, mainly dating from late nineteenth and twentieth century (thus examples of various styles of modernism). The sets of photographs were chosen to correspond to the context of the artworks. For example, the paintings by Cézanne had photographic equivalents that were provided by Machotka (1996) and consisted of photographs taken from very similar contemporary perspectives as Cézanne’s paintings. Likewise, the portraits depicted artists, politicians, etc., and their photographic counterparts consisted of photographs of the same individuals. A complete list of stimuli is shown in Table 1.

**Table 1:** **List of stimuli used in experimental task and explicit memory task**.

**I. EXPERIMENTAL STIMULI**	
**Four sets (8 matched pictures per set = 32 total images)**	
**Set 1: painted landscapes**	**Set 2: photographic landscapes**
L1: Cézanne “*Maison en provence-le vallon”*	Photo Machotka
L2: Cézanne *“Bassin et lavoir du Jas de Bouffan”*	Photo Machotka
L3: Cézanne “*Rochers à l’estaque”*	Photo Machotka
L4: Cézanne “*Le Pont de maincy”*	Photo Machotka
L5: Anton Koch “*Der schmadribachfall”*	Photo “*Der Schmadribachfall”*
L6: Ralf Scherfose “*Düne mit strandkorb”*	Photo “*Amrum”*
L7: Van Gogh “*Olivenbäume”*	Photo “*Olivenbäume in St. Remy”*
L8: Van Gogh *“Die Brücke von Langlois”*	Photo “*Brücke von Langlois”*
**Set 3: painted portraits portraits**	**Set 4: photographic portraits**
P1: Johannes Gruetzke *“Selbstbildnis”*	Photo “*J. Gruetzke”*
P2: Norbert Weck “*portrait Heinrich Böll”*	Photo “*Heinrich Böll”*
P3: Ralf Scherfose “*portrait prof. Dr. Alfred Gutschelhofer”*	Photo “*Gutschelhofer”*
P4: Johannes Heisig *“portrait Willy Brandt”*	Photo “*Willy Brandt”*
P5: Julian Schnabel “*Potrait of Olatz”*	Photo *“Olatz Schnabel”*
P6: Alice steel “Faith Ringgold”	Photo *“Faith Ringgold”*
P7: Norbert Wagenbrett “*Matthias Goerne”*	Photo “*Matthias Goerne”*
P8: Oskar Kokoschka “*Adele Astaire”*	Photo “*Adele Astaire”*
**II. EXPLICIT MEMORY TASK**	
**Four pairs of distracter images for each set (4 sets = 16 pictures)**	
**Painted and photographic landscapes**	***Similar pictures* (painted & photographic l)**
L1: Cézanne/Machotka “*Maison en Provence”*	C./M. *“Le Pigeonnier de Bellevue”*
L5: Anton Koch/Photo *“Der Schmadribachfall”*	A. Koch *“Reichenbachtal Mit Wetterhorn”*
L7: Van Gogh/Photo *“Olivenbäume”*	Van Gogh *“Weizenfeld mit u. Sonne”*
L8: Van Gogh/Photo *“Die Brücke von Langlois”*	Van Gogh *“Brücke von Trinquetaille”*
**Painted and photographic portraits**	***Similar pictures* (painted & photographic p)**
P1: Johannes Gruetzke *“Selbstbildnis”*	J. Gruetzke *“Richard von Weizsäcker”*
P5: Julian Schnabel *“Potrait of Olatz”*	J. Schnabel *“Portrait Nina Chow”*
P7: Norbert Wagenbrett *“Matthias Goerne”*	N. Wagenbrett *“Musiker Wolfram Dix”*
P8: Otto Kokoschka *“Adele Astaire”*	O. Kokoschka *“Alma Mahler”*

### Procedure

The study began with a pre-test in which the current cognitive state, as well as the stage of AD, were established with the MMSE. Also, participants were asked to indicate on an 11-point Likert-scale how much they are interested in art in general. In order to detect visual processing deficits, we employed a control task using colored drawings of everyday objects (Rossion and Pourtois, 2004). Participants were asked to rank-order eight objects according to their real-world size. This control task was tested in both sessions.

In the main experimental task, participants were asked to rank-order the stimuli for each of the four image sets. Each of the four sets of eight stimuli was put on a table in two rows in random order, and participants were asked to spatially sort them from left to right according to their esthetic preference. There was no time limit and positions could be changed until the person indicated that a solution was found. Participants were also told that no correct or wrong “answers” to any of the stimuli could be given. Afterward, the size-sorting control task was conducted.

Two weeks later, a second testing session was conducted. This session started with an explicit memory test. We performed a test of explicit recognition in which six pairs of images were shown, each comprising one old image (from the first session) plus a second distracter image, matched in terms of content. Distracter photographic images were chosen according to a definition by Konkle et al. (2010) to be conceptually similar, containing similar objects, regions, and color distributions. Distracter artworks were by the same artist, and of similar content and palette. Participants were asked to indicate which of the images they had seen 2 weeks previously.

After the explicit memory task, participants were tested on the rank-order preference task with the same stimuli as in the first session with the same procedures.

## Results

### Control task

The purpose of the real-world size ranking control task was to detect deficits in visual perception. We found that on average, 76% of the healthy elderly participants ordered objects according to their real-world size correctly, and only 19% interchanged one position. In the group of people with AD, 11% ordered the objects correctly, 33% interchanged one object, and 25% interchanged more than three. Participants in the experimental group clearly had some difficulty in solving the task. Some were already exhausted after the sorting task; some did not understand that this was not a preference task but rather that “sorting by size” was required; and some had already lost concentration, or started conversations regarding other issues. However, although this task could not be meaningfully analyzed, participants in the experimental group were generally able to name the depicted objects.

### Explicit memory task

Because the participants were more exhausted than initially expected, the recognition test comprised only four image pairs per set. In the experimental group the results of the forced choice task were at chance (*M* = 2.04, SD = 0.42), while in the control group the value was 3.03 (SD = 0.66). In other words, people in the control group recognized 76% of the images they had seen in the first testing session, the group with AD recognized 51% of them. A repeated measurement ANOVA with mean-correct recognition rates as dependent variable, and group (AD/control) as a between, and category (Portrait/landscape) and style (photography/art) factors, revealed a massive effect of group, with much higher performance for the control participants [*F*(1,31) = 28.75, *p* < 0.001, eta = 0.48] but only one trend for an interaction between group and style, with *F*(1,31) = 3.76, *p* = 0.062, eta = 0.11.

### Preference task

The stability of the ranks for preference was analyzed as in the Halpern et al. (2008) study, counting the change that each item had in the preference rankings between the two sessions. The added changes were computed to generate a change score. In particular, we first take the sum of the magnitude of the rank change for a given image set. For example, imagine if the rankings of the eight photo portrait images were in session 1 found to be (1,2,3,4,5,6,7,8), and in session 2 they were found to be (8,7,6,5,4,3,2,1). Let us consider the first four images in the session 1 ranking: they would change in rank by 7, 5, 3, and 1, respectively, giving a change score of 16. The last four images would have the same change scores (1, 3, 5, and 7, respectively), giving an overall summed change in ranking of 32 for this image set. Mean values of these scores were calculated for each category of images. Each of these change scores was then divided by the number of items (eight) in each set in order to make the scores comparable to the results of Halpern et al. (2008), who used this convention. Thus, the reported average rank change could vary in steps of 0.25, with a maximum value of 4 and a minimum value of 0. Higher values indicate lower consistency between first and second testing. Table 2 shows the mean change score values (and SD) in all four stimulus categories for AD patients and control participants.

**Table 2:** **Esthetic stability for AD patients versus controls for each stimulus category**.

	**AD**	**Controls**
Art portraits	1.86 (0.76)	1.53 (0.57)
Photo portraits	2.17 (0.66)*	1.55 (0.78)
Art landscape	1.53 (0.71)	1.75 (0.74)
Photo landscape	1.83 (0.84)	1.92 (0.79)

*Indicates that photo portraits showed significantly lower stability for AD patients compared to the control group (two-tailed *t*-test, *p* = 0.019).

We found a significant difference between the AD and controls only for the photographic portraits (*p* = 0.019, two-tailed *t*-test), with AD patients showing less stability. Preference stability did not show significant differences for any of the other three categories. These results imply that in comparison to controls, AD patients’ esthetic stability is impaired for images of natural faces but not for other types of painted images or photographs of landscapes.

All change-score means were in a range between 1.5 and 2.2, and the values seem to be more stable (smaller) in the control group for the portraits. We conducted an analysis of variance, with *art* (art-photograph) and *genre* (portrait-landscape) as within-factors and *group* (AD versus Controls) as between-factor, on the mean changes in preferences by participants as dependent variable. This analysis only revealed an interaction between *group* and *genre* [*F*(1,31) = 8.127, *p* < 0.01, eta = 0.21], but no other effect.

### Disease severity

Our range of MMSE scores allowed us to test for possible influences of disease severity on esthetic stability in AD (in Halpern et al., 2008, no AD patient scored lower than 12 on the MMSE, while in our sample, 7 patients in the AD group scored lower than 12 on the MMSE). An ANOVA analysis found no significant differences among the AD participants grouped by disease severity, nor were there significant differences between control group and each of the three disease severity groups. Mean stability scores (with SD) are listed in Table 3.

**Table 3:** **Esthetic stability as a function of disease severity for each stimulus type**.

	Art portraits	Photo portraits	Art landscapes	Photo landscapes
Severe	1.79 (0.47)	2.11 (0.52)	1.50 (0.84)	2.25 (0.98)
Moderate	2.00 (0.64)	1.95 (0.97)	1.50 (0.59)	1.60 (0.76)
Early	1.83 (1.17)	2.25 (0.65)	1.58 (0.77)	1.52 (0.59)

### Vector length analysis

We applied a second measure of esthetic stability based on vector length. Treating each subject’s rankings in each session as a vector, we took the *L*_2_-norm (Euclidean distance) of the difference in the vectors for the two sessions. This measure is in agreement with what was calculated above (average change score), which is effectively the *L*_1_-norm (city-block distance). All significant and insignificant effects found with the *L*_1_ metric were also found with the *L*_2_ metric.

### Possible confounds

There are some possible confounds in our study. In the landscape category, there were four paintings by Cézanne, so perhaps if viewers had similar esthetic judgments of all Cézanne images, this would artificially bias their responses to be more stable (assuming all works by Cézanne are judged similarly). However, we found that there was no significant difference in the change scores for the Cézanne images versus the other images in the landscape painting set (*p* = 0.91). Moreover, we see no difference in stability for controls versus AD patients for landscape photographs, which presumably have less influence of authorship. We observe the same also for portrait paintings, which were all painted by different artists.

Werheid and Clare (2007) suggest that intra-class similarity could play a role in face perception deficits in AD, thus the fact that the face photographs were rather similar in content could have influenced our results. However, the same is true of the portraits, and in any case there is substantial variety in poses, lighting, hairstyles, facial hair, gaze, direction, and clothing in the face photographs. The criticism of Werheid and Clare (2007) is more germane to face stimuli from highly standardized databases that are used in many studies. Nevertheless, it remains possible that intra-class similarity could have had some effect on our results.

The AD group was significantly older than the control group (89.5 versus 74.2). But despite the more advanced age of the AD group, it still showed similar stability compared to controls as described above.

Given that the AD group was mostly female (15/18), and if one supposes that face attractiveness is more salient in face photographs, one might expect that the AD group would tend to be biased to rate male faces as more attractive than female, which would tend to bias results in the face photograph task in the direction of greater stability. However, this does not appear to be the case because we find that the face photographs show significantly lower esthetic stability for the AD group. Moreover, only one face photograph showed greater stability in the AD group (P1, image of Johannes Gruetzke), and for this image the males showed greater stability than females. In addition, the control group was also biased toward females (10/15). However, gender effects deserve further study.

## Discussion

In general, our results replicate those of Halpern et al. (2008), though our results add substantial new understanding of esthetic perception in AD. We have provided evidence that people with both early- and later-stage Alzheimer’s-related dementia show similar degrees of esthetic stability. And we report that esthetic stability in AD is not different from controls for paintings and photographs of landscapes and paintings of faces, but not for photographs of faces. And in agreement with Halpern et al. (2008), AD patients showed chance-level explicit memory for the image groups, while the control group showed far better explicit memory for the images.

We are left with a basic question: Why are face photographs different? We propose a route to explaining the observed effects. First, a fundamental separation of esthetic beauty from biological beauty – perhaps one rooted in basic visual dimensions of art (Graham and Meng, 2011) – could play a role. In particular, human judgment of esthetic quality for art objects may rely on different perceptual and cognitive mechanisms compared to judgment of face or body attractiveness. Our result showing that landscape photographs do show esthetic stability suggests that this explanation cannot fully explain our findings. However, landscape photographs may have elements of artful posing that could be less prominent in face photographs (McManus et al., 2011). Such differences could contribute to the observed effects.

Let us also consider natural faces as a special stimulus class. When humans see a face *as a face* (not as a scene to be esthetically evaluated), we may think, do I know this person? And a person with AD, especially in earlier stages of the disease, might think, am I *supposed* to know who this person is? Such may not be the case for artwork – we have freedom to evaluate it along other criteria, which may result in more amodal or less task-directed processing compared to faces.

In one of the very few previous studies that has bearing on the question of why stability for face photographs is different, Hönekopp (2006) showed that esthetic stability for face photographs in a sample of healthy young people (mean age ∼24 years) is very high over a 1-week span. Therefore, we would expect a high baseline even for older adults. And as noted above, some evidence indicates that recognition of familiar faces appears relatively intact compared to recognition of familiar scenes early in the progression of AD (Cheng and Pai, 2010). Face-matching ability is also somewhat spared in those with established AD (Kurylo et al., 1996). But without explicit memory for faces, as shown in our study, AD patients may experience cognitive interference, which impairs their esthetic stability. We therefore refer to this explanation of our results as the *cognitive interference hypothesis*. In this view, our results are consistent with the idea that partially spared memory for the familiarity of faces generates a cognitive conflict such that patients may suspect they should be able to name or recognize a face, but cannot.

Why, then, do portrait paintings yet show similar esthetic stability in AD and in controls? A partial answer can perhaps be found if we consider other previous results. Kurylo et al. (1996) found that AD patients’ performance on the Mooney Face Test (a measure of face/non-face discrimination using binarized images) was poor compared to their performance on other visual perception tasks (e.g., a spatial position task). Thus, decreased ability to recognize stylized faces *as faces* (or as *specific faces*) could enable portrait paintings to be evaluated more easily on basic esthetic grounds, with less interference of face detection and recognition systems.

Our method does not elucidate the process by which AD patients evaluate esthetic qualities, nor does it prove that people with AD are truly judging esthetic quality in the same way as controls despite their comparable performance on the rank-order task. The situation is made all the more complex because if people with AD do possess esthetic perception much like that of healthy adults, we must explain how this is possible given that brain areas that seem to underlie normal esthetic perception appear to be damaged in AD. We speculate that apparent damage may not be so severe as to obliterate basic esthetic responses in AD, and that sub-cortical systems could help individuals compensate. Future studies – including those concerned with brain lesions – may help uncover what operations are necessary at a minimum to make esthetic judgments. This taps into an important debate on whether the multiple facets of the esthetic sense are essentially based on early sensory or later cognitive processes (Leder, 2013). In any case, we believe this is an important area for future research.

One might wonder why we would expect esthetic perception to be stable in the first place. However, our method probes preference of a set of stimuli *relative to themselves* over a 2-week span – which we (Halpern et al., 2008) presume to be stable. Images are also presented in the same context in both sessions. We certainly might expect instability in cases where new and old stimuli are mixed, as in Park et al. (2010). But even in the Park et al. (2010) study, preference shifts due to novelty/familiarity manipulations in one session were erased after a 1-week interval. Thus it would appear unlikely that judgments of preference should be affected by exposure (including repeated exposure) to other images during the interval. Nevertheless, other experiences in the interval could play a role, and this is an important but so far neglected question. It remains to be seen whether preference for a standard set of stimuli is malleable in healthy adults and other populations, and what could cause such shifts.

### Future studies

We argue that the response of AD patients in the present experiments could be considered a “pure” form of visual esthetic perception, one that relies solely on image content. Although we do not want to minimize the often tragic cognitive decline of those with AD, and we would not claim this is a kind of silver lining in this condition, we would advocate that the present study and future studies of AD be considered as novel and uniquely important form of evidence regarding human esthetics. If this interpretation is correct, one conclusion we can draw is that the current results support the idea that there is a fundamental dichotomy between biological and artistic esthetics for humans as a species.

However, as we have noted, an important question that will require further research in order to put the current study and Halpern et al. (2008) in context concerns the baseline esthetic stability in younger people and in other populations. While healthy older adults and AD patients mostly show similar esthetic stability, we currently do not have a well-established benchmark for humans as a whole. We are not aware of any studies that have examined this aspect of esthetics for artistic images, although a handful of papers have tested the stability of preference over time for other images (e.g., Hönekopp, 2006). We are currently undertaking a suite of experiments in a variety of populations to establish benchmarks for esthetic stability, taking account of contextual factors.

Finally, we propose that a main requirement for activating esthetic responses in AD could be for art images to be perceived as such. If true, this insight could help guide the development of novel therapies, particularly those involving art viewing. Future experiments examining the potential for art images to be used as avenues for lessening the severity of disease symptoms could therefore be fruitful. A deeper understanding of visual esthetics in AD could also assist in the development of more effective signage in care facilities by leveraging stimuli that promote esthetic stability.

## Conclusion

Our results demonstrate that people with AD have esthetic stability for artistic images mostly like that of healthy older controls, and that this stability is roughly the same for early- and later-stage AD patients. This stability contrasts with the lack of explicit memory for the stimuli in the AD group. However, face photographs show decreased esthetic stability in AD patients compared to controls. Given these results, we conclude that, for people with Alzheimer’s-related dementia, esthetic perception of artistic images represents an “island of stability” in a condition that in most other respects causes profound cognitive disruption. We have proposed interpretations of our results that give a novel perspective on face perception in AD, and also on perceptual esthetics. We hope that our findings will contribute to new approaches to therapy for AD patients.

## Conflict of Interest Statement

The authors declare that the research was conducted in the absence of any commercial or financial relationships that could be construed as a potential conflict of interest.
